# Clinical significance of hyper-IGA in a pediatric laboratory series

**DOI:** 10.1186/1546-0096-12-S1-P170

**Published:** 2014-09-17

**Authors:** Serena Ifmach Pastore, Valentina Copetti, Carlo De Pieri, Oriano Radillo, Andrea Taddio, Alessandro Ventura, Alberto Tommasini

**Affiliations:** 1 University of Trieste, Institute of Maternal and Child Health – IRCCS- Burlo Garofolo, Trieste, Italy; 2 Department of Advanced Diagnostic and Clinical Trials, Institute of Maternal and Child Health – IRCCS- Burlo Garofolo, Trieste, Italy

## Introduction

Very high levels of one or more classes of immunoglobulins can be found in children with various clinical conditions. Diagnostic protocols have been developed for defined forms of IgG, IgM or IgE hypergammaglobulinemia, which could be the expression of both chronic inflammatory disease and primary immunodeficiency syndromes. In contrast, except for well described conditions such as IgA nephropathy and Henoch Schönlein Purpura, much less is known about hyper-IgA.

## Objectives

We analyzed and discussed the diagnostic significance of very-high IgA levels in the clinical practice of a tertiary care children hospital.

## Methods

We collected all IgA determinations performed on children aged less than 16 years at the laboratories of Immunopathology of the Institute for Maternal and Child Health, IRCCS Burlo Garofolo, from 2009 to 2013. IgA values greater than three standard deviations above the mean, based on the local reference values for three age groups, were considered as “hyper-IgA” (0 – 12 months, IgA > 113 mg/dl; 1 – 3 years, IgA > 225 mg/dl; 3-16 years, IgA > 368 mg/dl). For subjects with repeated hyper-IgA measures on different determinations, only the first value was recorded. To estimate the burden of diseases associated with hyper-IgA, a control age-matched group of 200 subjects with normal IgA values was randomly selected from the same laboratory series. Physicians who cared for each patient were asked to fill out a questionnaire regarding the clinical diagnosis and the interpretation of increased values of IgA. In addition, the levels of IgG and IgM greater than 2 standard deviations above the mean were recorded as well, where available. Subjects whose clinical records were not available were excluded from the analysis.

## Results

A total of 12650 measurements of serum IgA were performed during the period of the study on 6364 subjects. Ninety-one subjects (48 males, 43 females, mean age 9.1 years) had one or more hyper-IgA values (mean IgA value 427.1 mg/dL, range 114 – 1051 mg/dl). Clinical records were available for 83/91 subjects with hyper-IgA and 173/200 age-matched controls with normal IgA. Most subjects with hyper-IgA (73.5%) suffered from a severe immune defect, a chronic rheumatic disease or an inflammatory bowel disease, while these conditions were very rare in a control group with normal IgA values (8%).

## Conclusion

The potential role of hyper-IgA in pediatric diagnostics may be greater than usually recognized, even if this high prevalence of serious disease with high IgA may not apply to general population. We suggested that hyper-IgA in children should always rise the suspicion of a serious immune defect, a chronic rheumatic disease or an inflammatory gastrointestinal disorder.

## Disclosure of interest

None declared.

